# Reverse-Phase Phosphoproteome Analysis of Signaling Pathways Induced by Rift Valley Fever Virus in Human Small Airway Epithelial Cells

**DOI:** 10.1371/journal.pone.0013805

**Published:** 2010-11-03

**Authors:** Taissia G. Popova, Michael J. Turell, Virginia Espina, Kylene Kehn-Hall, Jessica Kidd, Aarthi Narayanan, Lance Liotta, Emanuel F. Petricoin, Fatah Kashanchi, Charles Bailey, Serguei G. Popov

**Affiliations:** 1 National Center for Biodefense and Infectious Diseases, George Mason University, Manassas, Virginia, United States of America; 2 Virology Division, United States Medical Research Institute of Infectious Diseases, Fort Detrick, Maryland, United States of America; 3 Center for Applied Proteomics and Molecular Medicine, George Mason University, Manassas, Virginia, United States of America; Hallym University, Republic of Korea

## Abstract

Rift valley fever virus (RVFV) infection is an emerging zoonotic disease endemic in many countries of sub-Saharan Africa and in Egypt. In this study we show that human small airway epithelial cells are highly susceptible to RVFV virulent strain ZH-501 and the attenuated strain MP-12. We used the reverse-phase protein arrays technology to identify phosphoprotein signaling pathways modulated during infection of cultured airway epithelium. ZH-501 infection induced activation of MAP kinases (p38, JNK and ERK) and downstream transcriptional factors [STAT1 (Y701), ATF2 (T69/71), MSK1 (S360) and CREB (S133)]. NF-κB phosphorylation was also increased. Activation of p53 (S15, S46) correlated with the increased levels of cleaved effector caspase-3, -6 and -7, indicating activation of the extrinsic apoptotic pathway. RVFV infection downregulated phosphorylation of a major anti-apoptotic regulator of survival pathways, AKT (S473), along with phosphorylation of FOX 01/03 (T24/31) which controls cell cycle arrest downstream from AKT. Consistent with this, the level of apoptosis inhibitor XIAP was decreased. However, the intrinsic apoptotic pathway marker, caspase-9, demonstrated only a marginal activation accompanied by an increased level of the inhibitor of apoptosome formation, HSP27. Concentration of the autophagy marker, LC3B, which often accompanies the pro-survival signaling, was decreased. Cumulatively, our analysis of RVFV infection in lung epithelium indicated a viral strategy directed toward the control of cell apoptosis through a number of transcriptional factors. Analyses of MP-12 titers in challenged cells in the presence of MAPK inhibitors indicated that activation of p38 represents a protective cell response while ERK activation controls viral replication.

## Introduction

Rift Valley fever virus (RVFV) is a highly-pathogenic arthropod-borne *Phlebovirus* of the *Bunyaviridae* family that infects a wide range of vertebrate hosts. In humans RVFV infection can lead to encephalitis, retinitis, or fatal hepatitis associated with hemorrhagic fevers. In ruminants it is associated with high mortality rates, abortion, and fetal deformities [1], [2]. RVFV infection is an emerging zoonotic disease endemic in many countries of sub-Saharan Africa and in Egypt. During the last decade the number of devastating outbreaks increased progressively [3], [4]. Although the major route of RVFV entry into the host is through mosquito bites, some evidence indicates that aerosol route of infection can be highly effective. Laboratory workers have acquired RVFV supposedly after inhalation of infectious aerosols generated by careless handling of infected tissues [5]. Also, several animal species have been shown experimentally to be highly susceptible to airborne RVFV [5], [6]. From this point of view, RVFV is considered as a potential biothreat. However, the frequency of airborne RVFV transmission under natural conditions remains unknown and interaction of the virus with the potential cell targets in the respiratory tract has not been studied.

The information on the molecular biology of RVFV and its interaction with host cells is limited. RVFV has a tripartite single-stranded RNA genome consisting of large (L), medium (M), and small (S) segments [7]. The L and M segments are of negative polarity and express, respectively, the RNA-dependent RNA polymerase L and the precursors to the glycoproteins Gn and Gc. Gn and Gc cleavage also generates a nonstructural protein (NSm) that has been recently identified as a suppressor of virus-induced apoptosis [8]. The S segment utilizes an ambisense strategy and encodes the nonstructural protein NSs in genome orientation and the nucleoprotein N in antigenome orientation. RVFV NSs protein is not essential for virus replication in cell culture [9], while it works as a major viral virulence factor in infected animals [10]. NSs is expressed early in virus infection prior to viral RNA replication. It inhibits the induction of interferon beta [11], suppresses the host innate immune system by downregulation of PKR through its degradation in proteasomes and inhibition of eIF2-alpha phosphorylation [12], along with the general inhibition of cellular transcription [13].

Phosphorylation plays a key role in regulating many signaling pathways. Cell fate decisions in response to extracellular agents, including pathogenic invaders, are commonly mediated by phosphorylation-regulated signaling cascades that transduce signals into stimulus-specific actions, such as changes in gene expression pattern. Generally, both proapoptotic and prosurvival pathways are activated during viral replication. The possibility to interfere with virus replication either by enhancing antiviral signaling or by inhibiting proviral signaling can open a new avenue to antiviral treatments and prophylaxes. For example, in the proof-of-concept studies the inhibitors of Raf/MEK/ERK mitogenic kinase cascade and the IKK/NF-kB module reduce influenza virus titer in the lungs of infected mice after local aerosol administration into the trachea [reviewed in 14]. However, in the case of RVFV this potential opportunity has not been previously explored because of the limited knowledge of the intracellular events during infection. We therefore characterized the phosphoprotein signaling by RVFV in the human small airway lung epithelial cells (HSAECs) as an *in vitro* model relevant to aerosol exposure. Using a high-throughput reverse-phase phosphoproteome analysis during the time course of infection of HSAECs with the virulent wild-type RVFV strain ZH-501 we identified changes in major prosurvival and proapoptotic pathways controlled by the virus. Our analyses suggest a number of phosphoproteins as potential points of pharmacological interventions.

## Materials and Methods

### Reagents

The ZH-501 strain of RVFV was isolated in 1977 from the blood of a 10-year-old Egyptian girl, who had a fatal RVFV infection [15]. This strain was passed twice in fetal rhesus monkey lung cells and once in Vero (African green monkey kidney) cells before use in this study. The attenuated RVFV MP-12 strain was a kind gift from Dr. Sina Bavari, United States Army Medical Research Institute of Infectious Diseases. The RVFV MP-12 was propagated and titrated using Vero cells. Experiments with live ZH-501 virus were carried out in the BSL-3 containment lab at the Virology Division of the United States Army Medical Research Institute of Infectious Diseases, MD, USA. Experiments with the MP-12 strain were carried out in the BSL-2 environment at the Biomedical Research Laboratory, George Mason University, Manassas, VA.

Cell culture reagents were from CellGro (Herndon, VA). Antibodies against total and phosphorylated forms of the following proteins used for reverse-phase protein microarrays (RPMA) and Western blots were from Cell Signaling Technology (Beverly, MA) and were used at the dilutions indicated: 1∶2000 for ERK1/2 (T202/Y204), 1∶200 for STAT1 (Y701), 1∶50 for Caspase-3 cleaved (D175), 1∶100 for Caspase-7 cleaved (D198), 1∶50 for Caspase-6 cleaved (D162), 1∶1000 for p53 (S46), 1∶50 for SAPK/JNK (T183/Y185), 1∶1000 for p53 (S15), 1∶200 for p90RSK (S380), 1∶100 for HSP27, 1∶50 for MSK1 (S360), 1∶50 for p38 (T180/Y182), 1∶500 for MEK1/2 (S217/221), 1∶100 for STAT3 (S727), 1∶100 for ATF-2 (T69/71), 1∶50 for NF-kappaB p65 (S536), 1∶400 for CREB (S133), for IRS-1 (S612), 1∶1000 for Puma, 1∶250 for FOX 01/03a (T24/32), 1∶250 for Src (Y527), 1∶1000 for Bim, 1∶100 for Akt (S473), 1∶100 for XIAP, 1∶100 for LC3B, 1∶50 for Cyclin D1, 1∶100 for FADD (S194), 1∶250 for GSK-3 alpha/beta (S21/9), 1∶500 for PKR (T451), 1∶50 for Beclin-1, 1∶100 for mTOR (S2481), 1∶500 for PTEN (S380), 1∶100 for total p38, 1∶50 for p70S6 Kinase (S371), 1∶50 for AMPKalpha1 (S485), 1∶500 for Bax, 1∶100 for Bad (S136), 1∶100 for Elk1 (S383), 1∶1000 for Bid, 1∶500 for total SAPK/JNK, 1∶200 for c-Raf (S338), 1∶100 for caspase-9 cleaved (D315); 1∶100 for caspase-9 cleaved (D330), 1∶500 for Atg 5, 1∶50 for FAK (Y397), 1∶500 for PP2A A Subunit, 1∶100 for ASK1 (S83), 1∶200 for eNOS (S1177), 1∶50 for b-Raf (S445), 1∶200 for HSP90 (E289), 1∶100 for total AKT, 1∶1000 for total ERK1/2, 1∶100 for Bcl-2 (T56), 1∶100 for p27(T187),1∶100 for Src family (Y416), 1∶200 for beta actin, 1∶100 for total p53, 1∶100 for Jak1 (Y1022/1023), 1∶50 for HSP70, 1∶1000 for total PKR. The secondary HRP-labeled goat anti-rabbit antibody was from Cell Signaling Technology (Beverly, MA) and was used at the dilution 1∶10000 for Western blot. The antibody against the viral antigen was from ProSci, Inc. (Poway, CA). It was raised against a peptide corresponding to a fragment near the center of the protein precursor translated from the M segment. It therefore detects both the precursor and the glycoprotein G1.

### Challenge of HSAECs with RVFV

HSAECs (Cambrex Inc., Walkersville, MD) from anonymous donor were grown according to the vendor's protocol in Ham's F12 medium supplemented with nonessential amino acids, pyruvate, β-mercaptoethanol and 10% fetal calf serum (FCS) at 37°C, 5% CO_2_. The cells were adapted to these culture conditions during four passages and then were used for the preparation of the frozen stock. For reverse phase protein (RPMA) microarray experiments, confluent HSAECs at 10^6^ cells per well in 6-well plates were challenged with RVFV ZH-501 at the multiplicity of infection (MOI) of 0.0002 and 0.002. 100 µl of RVFV suspension in a diluent (10% heat-inactivated FBS in Medium 199 with Earle's salts, NaHC0_3_ and antibiotics) containing 10^3.3^ and 10^2.3^ PFU of RVFV (MOI of 0.002 and 0.0002, correspondingly) or diluent were added to each well. Cells were incubated for 1 h at 37°C in the CO_2_ incubator and then 3 ml of HSAECs medium were added to each well and the plates returned to the CO_2_ incubator. At 0, 5, 24, 30, 48, 72 h post virus infection (h.p.i.), supernatant was removed, the cells were mixed with 500 µl of the lysis buffer, and immediately boiled for 10 min. The lysis buffer consisted of 1∶1 mixture of T-PER Reagent (Pierce, IL) and 2×Tris-glycine SDS sample buffer (Novex, Invitrogen, CA) in the presence of 2.5% β -mercaptoethanol, and protease and phosphatase inhibitors (1× Halt cocktail, Pierce). Lysed samples were stored at −80°C. Three independent cell challenge experiments were performed for the RPMA and Western blot analyses.

### RPMA and Western blot analyses

Approximately 30 nl of each sample (equivalent to the amount of material from 40 lysed cells) were arrayed onto nitrocellulose slides (Whatman, MA) by direct contact printing using a high-resolution 2470 arrayer (Aushon Biosystems, Billerica, MA). Samples were printed as duplicates of an eight-point two-fold dilution series. The linear range of each dilution curve was then used for quantitative data comparisons between samples. Slides were stored at −20°C prior to detection. To estimate the total protein amount in each spot, several randomly selected slides were stained with Sypro Ruby Protein Blot Stain (Molecular Probes, Eugene, OR) and visualized on a Fluorchem imaging system (Alpha Innotech, San Leandro, CA). Slides were stained on a Dako Autostainer with specific antibodies using a biotin-linked peroxidase-catalyzed signal amplification (Dako, CSA kit). Specifically, the arrayed slides were placed into Re-Blot solution (Chemicon, Temecula, CA) for 15 min, washed two times for 5 min each in PBS, placed into I-Block solution (Applied Biosystems, Foster City, CA) in PBS with 0.1% Tween-20 for at least 2 h, and then immunostained using an automatic slide stainer (Autostainer, Dako Cytomation) and the manufacturer-supplied reagents. Briefly, the slides were incubated for 5 min with hydrogen peroxide, rinsed with high-salt Tris-buffered saline (CSA Buffer, Dako) supplemented with 0.1% Tween-20, blocked with avidin block solution for 10 min, rinsed with CSA buffer, and then incubated with biotin block solution for 10 min. After another CSA buffer rinse, 5-min incubation with protein block solution was followed by air drying. the slides were incubated with either a specific primary antibody diluted in Dako Antibody Diluent or, as a control, with only Dako Antibody Diluent for 30 min. Every antibody underwent extensive validation ensuring a specific detection of the antigenic protein as a single band on the Western blot of cell lysate. The slides were washed with CSA buffer and incubated with a secondary biotinylated goat anti-rabbit IgG H1L antibody (1∶7500) (Vector Labs, Burlingame, CA) for 15 min. Slides were incubated with diaminobenzidine chromogen diluted in Dako diaminobenzidine diluent for 5 min, washed in deionized water and imaged using a Umax PowerLook III scanner (Umax, Dallas, TX) at 600 dpi. The images were analyzed with software AlphaAse FC (Alpha Innotech). For each antibody, the average pixel intensity value for negative control (staining with second antibody only) was subtracted from the average pixel intensity value for specific antibody and then normalized by the corresponding average value of the total protein intensity. Each sample dilution curve in each experiment was fitted with a nonlinear approximation equation and statistically evaluated using GraphPad Prism ver5 software (Graphpad Software, CA). The dilution of different samples chosen within the linear response interval was used to calculate relative analyte concentrations and their 95%-confidence intervals. The RPMA experiments were repeated 3 times to ensure consistent results.

Signaling proteins demonstrating differences between control and challenged cells were further tested with Western blots using 15 µl of cell lysates, 1∶1000-diluted primary antibodies, and 1∶10000-diluted secondary antibody (goat anti-rabbit). Proteins were transferred to a nitrocellulose membrane using iBlot Gel Transfer Device (Invitrogen). The membranes were developed with SuperSignal West Femto Maximum Sensitivity Substrate (Pierce), and band intensities were measured with a Molecular Imager ChemiDoc XRS System (Bio-Rad, CA). The intensities of bands were calculated relative to untreated controls after densitometry using the QuantityOne 4.6.5 program (Bio-Rad). All measurements were performed in triplicate. Data are represented as mean values with 95%-confidence intervals (two-tail t-test, *α* = 0.05, *n* = 3).

### MP-12 infection and inhibitor studies

For Western blot analyses, HSAECs were grown in HAM's F-12 medium for 100% confluence in 12 well plates. Next day after confluency the cells were rinsed with PBS twice and challenged at MOIs of 0.002 and 2 at 37°C, 5% CO_2_ for 1 h with 400 µl RVFV MP-12 propagated and titrated using the Vero cells. After 1 h the virus was removed, cells were rinsed with PBS twice and 1 ml of HAM's F-12 medium was added to each well. The infected cells were incubated at 37°C, 5% CO_2_ for 24, 48 and 72 h. After each time point the cells were rinsed with PBS twice, lysed in 250 µl of lysis buffer used in the RPMA experiments, and boiled for 10 min.

For the inhibitor studies, HSAECs were seeded at 50,000 cells per well in a 96-well plate. The inhibitors (from Calbiochem) were dissolved in DMSO and added to the cells for 5 h prior to infection. A DMSO-only control was included and the final concentration of DMSO was 0.1%. Final concentrations of inhibitors were: 1 µM for p38 inhibitor SB203580, 10 µM for JNK inhibitor II and 10 µM for MEK1/2 Inhibitor III. The latter is known to specifically inhibit phosphorylation of ERK1/2. Medium was removed and cells were infected with RVFV MP-12 at MOI of 0.002 in 25 µl of medium. After one hour of infection, fresh medium containing inhibitors was added to cells. Supernatants were collected at 48 h.p.i., viral RNA extracted using Ambion's MagMAX™-96 Viral RNA Isolation Kit, and RNA analyzed by q-RT-PCR. RVFV q-RT-PCR assays were performed with a titration of purified viral RNA to establish a standard curve. The primers and probe used were originally described by Drosten *et al.* [1]. Assay conditions were as follows: 0.4 µM primers, 0.5 µM FAM-probe, 1 mM additional MgSO4, 0.4 µl Rox reference dye, 1.0 µl RNA UltraSense™ Enzyme Mix, 4.0 µl RNA UltraSense™ 5× Reaction Mix. Reactions were performed in a 20 µl volume using an ABI 7000 Real Time PCR instrument. Cycling conditions were 50°C for 15 min, 95°C for 2 min, followed by 40 cycles of 95°C for 15 s and 60°C for 30 s.

## Results

### RVFV infection has a profound influence on phosphoprotein signaling in HSAECs

**Figure 1:**
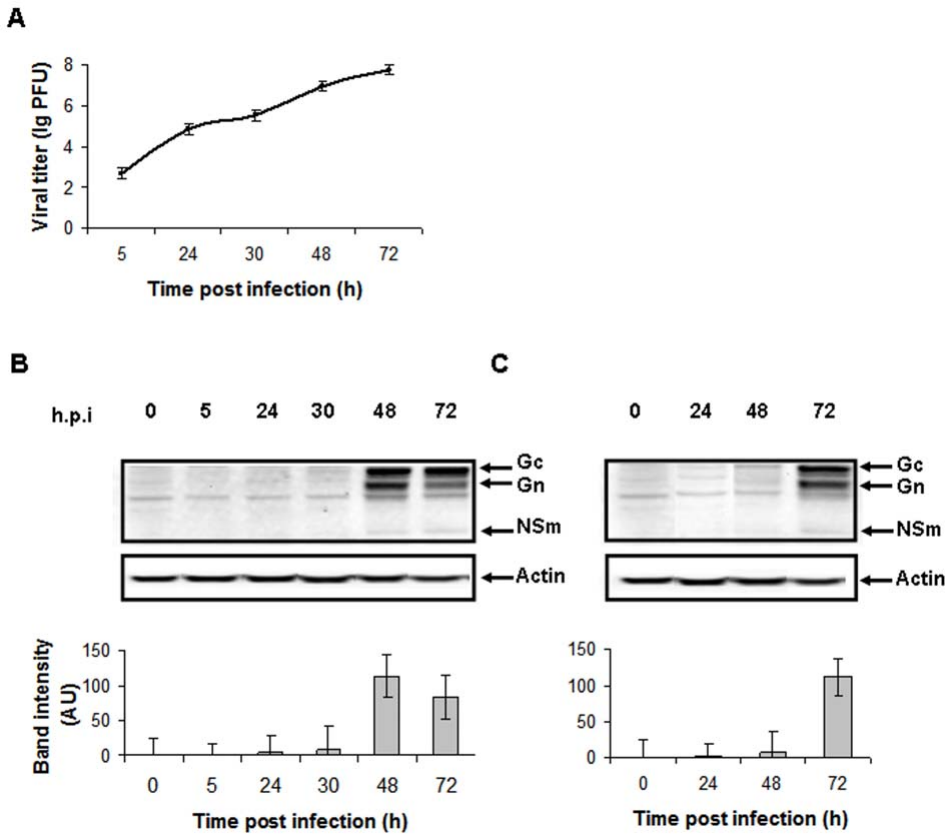
Quantitative analysis of the newly-formed virus released from HSAECs into culture supernatants. A. Plaque assay data are presented as lg (PFU/ml) in supernatants of RVFV-infected HSAECs for MOI of 0.002. B, C. Western blot of lysed cells using antibodies against peptide from the middle of RVFV M segment and against actin (B, for MOI of 0.002 and C, for MOI of 0.0002). The amount of cells loaded was 1.4×10^4^ cells per well. Band intensities (arbitrary units) of viral proteins above the background detected in uninfected cells are shown as graphs below of Western data. The positions of Gc, Gn and NSm proteins translated from the M-segment are shown with arrows.

To study host cell signaling by protein phosphorylation during RVFV infection, we used a model of HSAECs challenged with the virulent strain of RVFV (ZH-501). We hypothesized that this model would faithfully reflect signaling induced by inhaled RVFV in the infected lung epithelium. HSAECs were grown to confluency in 6-well plates. One day later the cells were infected with the virus at MOIs of 0.0002 and 0.002. The MOIs were chosen to avoid rapid apoptosis of cells and resemble the low-dose *in vivo* infection by mosquito bites or inhalation. RVFV was able to cause a productive infection of HSAECs as detected by a progressive cytopathic effect upon visual examination of cell monolayer morphology at different times post infection (data not shown). Cytopathic effect (rounding up and dislodging of cells from the plastic substrate) was first observed at 72 h. Quantitative analysis of the newly-formed virus released from HSAECs into culture supernatants was performed by plaque assay (Fig. 1a). In addition, the levels of intracellular virus antigen during the period from 5 to 72 h post infection (h.p.i.) were tested by Western blot of cell lysates using antibody specific to the viral proteins Gc, Gn and NSm (Fig. 1b, c). Concentration of viral antigen reached maximum at 48 and 72 h.p.i. (for MOI of 0.002 and 0.0002, respectively). Collectively these results indicate that active infection was observed in primary lung epithelial cells at both MOIs.

**Figure 2:**
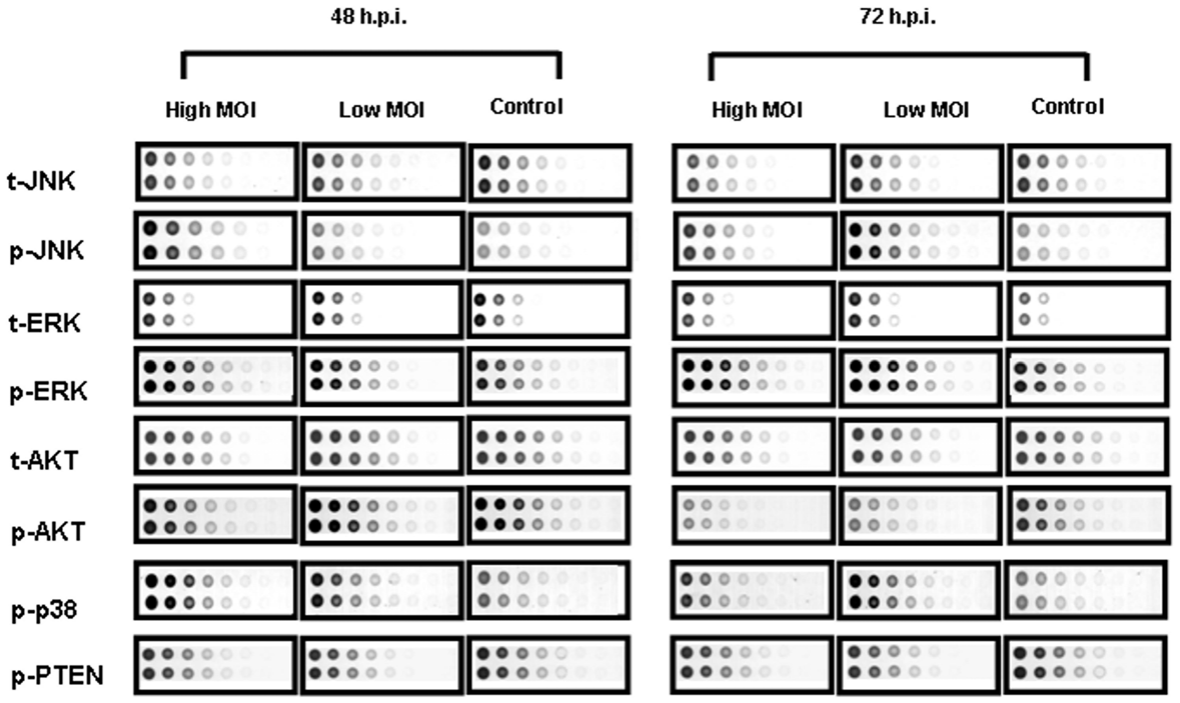
Example of RPMA slides stained with antibodies against total and phosphorylated forms of SAP/JNK, ERK and AKT and against phosphorylated form of p-38 and PTEN. Portions of slides stained with antibodies against total and phosphorylated protein forms are shown for untreated (Control) and RVFV-infected HSAECs at 48 and 72 h.p.i. Low and high MOI correspond to 0.0002 and 0.002, respectively. Each sample along with eight 2× serial dilutions was printed in duplicate. Prefixes *t* and *p* indicate the total and phosphorylated proteins, correspondingly.

For phosphoproteomic analysis the cell lysates were printed onto nitrocellulose membrane slides. Each slide was then probed with one of 60 different antibodies specific against phosphorylated or total forms of signaling proteins. The antibodies were selected to monitor the molecular networks involved in host responses most likely affected by virus exposure, namely survival, apoptosis, inflammation, growth, differentiation, and immune response. This RPMA technique was extensively validated in our previous studies [16] with regard to specificity of antibodies, sensitivity, and accuracy of phosphoprotein detection in cell lysates. As an example, slides stained with antibody against total and phosphorylated form of SAP/JNK, ERK and AKT and against phosphorylated form of p-38 and PTEN are shown in Fig. 2. All samples for untreated and RVFV-infected HSAECs from different time points were printed on the same slide. Boxed dots show untreated and RVFV-infected HSAECs at 48 and 72 h.p.i. at MOI of 0.002 (high) and MOI of 0.0002 (low). Significant changes are seen in the levels of phosphorylated forms of SAP/JNK, p38, ERK and AKT for RVFV-infected cells in comparison with untreated cells, whereas total forms of these proteins demonstrate only subtle changes. PTEN illustrates the protein which phosphorylation was not affected by RVFV infection.

**Figure 3:**
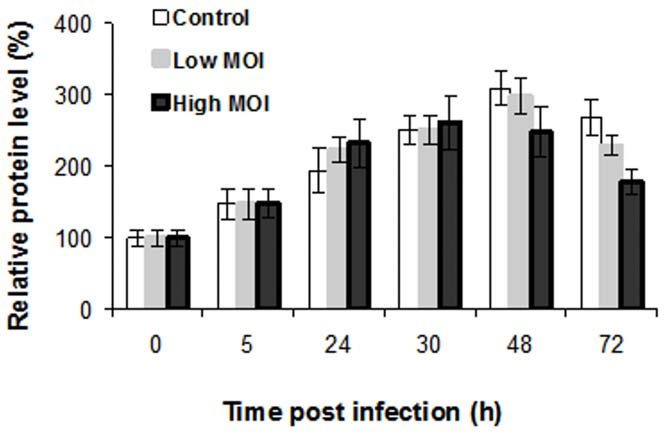
Total level of cellular proteins in RVFV-infected HSAECs. Slides stained with Sypro Ruby and normalized relative to uninfected HSAECs at 0 h.p.i. Each sample of 40 cells per dot along with seven 2× serial dilutions was printed in duplicate. Black and gray bars illustrate the MOI of 0.002 and 0.0002, correspondingly. Data represent mean values and confidence intervals of two-tail t-test (*α* = 0.05, *n* = 3).

In order to carry out quantitative analyses of RPMA data, the average total level of cellular protein at every time point was determined by staining with Sypro Ruby Protein Blot Stain of four randomly selected slides throughout the print run (Fig. 3). Similar levels of total protein were detected in infected and uninfected cells at the same time points post infection, likely reflecting cell growth without a detectable lysis up to 48 h.p.i. However, due to the progressive cytopathic effect of the virus, cell lysis became evident at 72 h.p.i. Since the RPMA analysis reflects the levels of intracellular proteins, all data were normalized to the total level of cellular proteins. The normalized values allowed direct comparison of results during the time course of infection.

**Figure 4:**
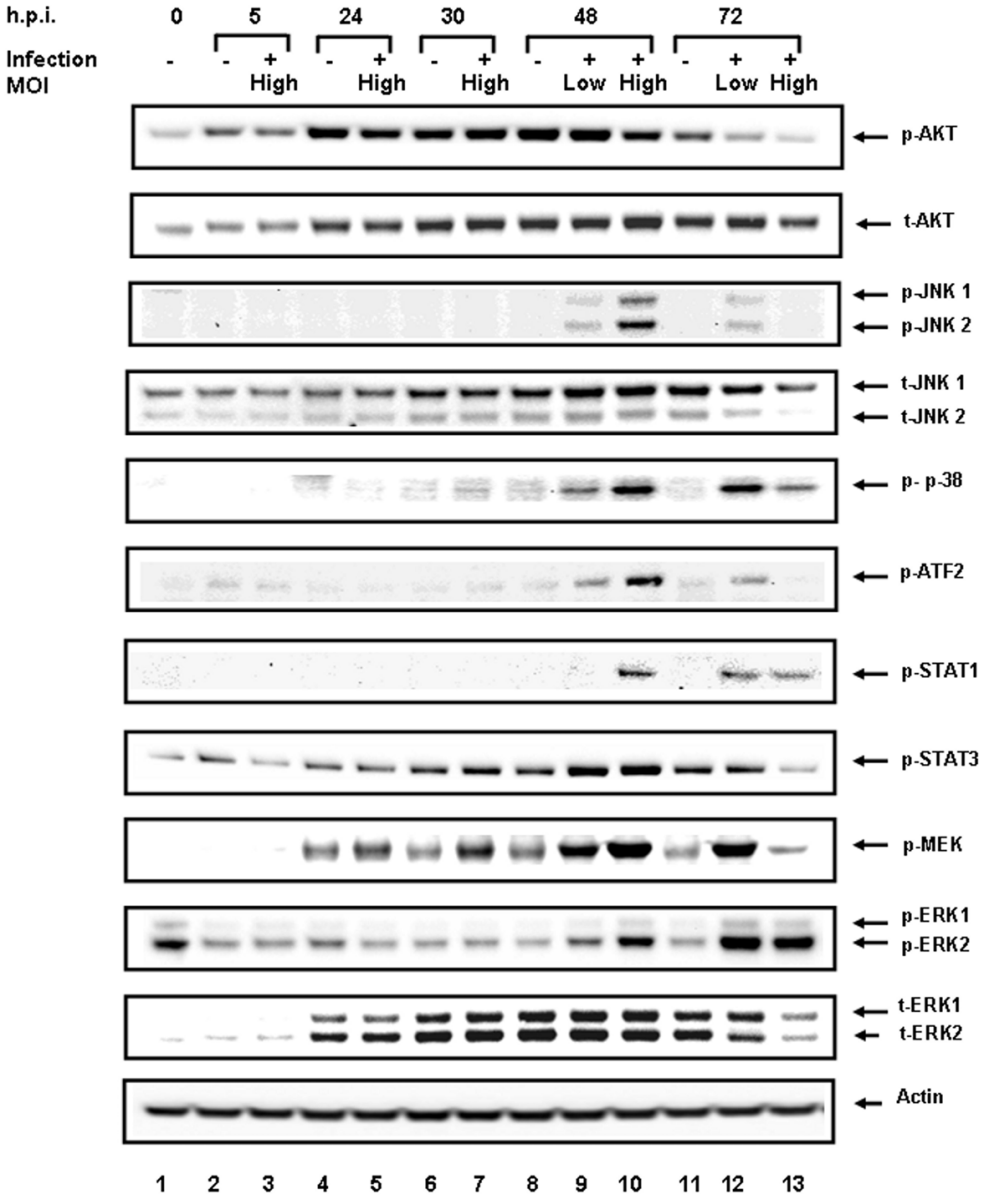
Confirmation of RPMA data by Western blot using antibodies against phosphorylated and total forms of several proteins. Western blots were performed on two independent experiments. The RVFV-infected HSAECs with MOI of 0.002 and 0.0002 at corresponding time points are indicated with a + sign. Samples 1, 2, 4, 6, 8 and 11 are from untreated cells at 0, 5, 24, 30, 48 and 72 h.p.i. and samples 3, 5, 7, 10 and 13 are RVFV-infected HSAECs with MOI of 0.002 at 5, 24, 30, 48 and 72 h.p.i. Samples 9 and 12 correspond to the cells infected with MOI of 0.0002 at 48 and 72 h.p.i.

**Figure 5:**
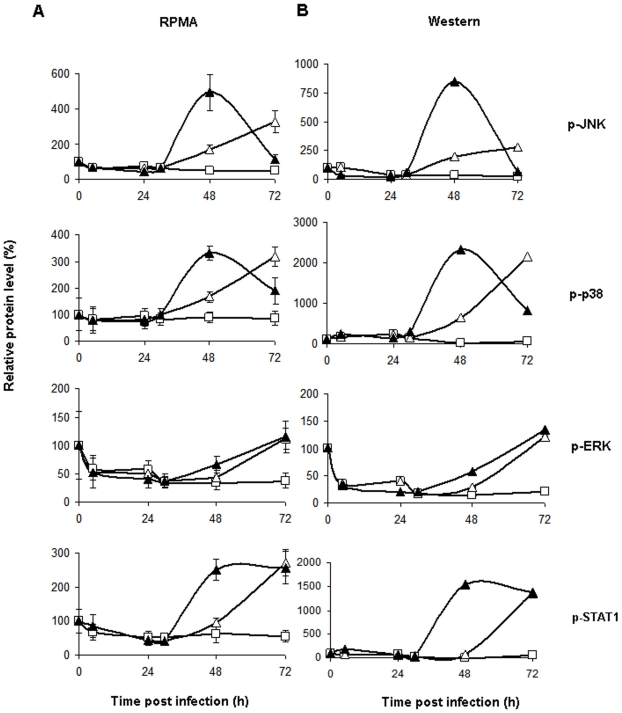
Comparison of RPMA and Western blot data. Left panels (A) show the RPMA data. Right panels (B) show Western blot confirmatory data. X axis labels, time post infection (h.p.i.); Y axis labels, protein level relative to untreated cells (%) at 0 h.p.i. Open triangles, closed triangles and open squares correspond to the low MOI, high MOI and uninfected cells, respectively. RPMA data represent mean values and confidence intervals of two-tail t-test (*α* = 0.05, *n* = 3). Western blot data represent average of two independent experiments.

Of the 60 tested signaling proteins, the relative levels of 27 phosphorylated proteins and activated protein forms (such as cleaved caspases) showed statistically significant (*p<*0.05) differences compared to uninfected cells at 48 and 72 h.p.i. (Table 1). Earlier time points did not demonstrate statistically significant differences between uninfected and RVFV-infected HSAECs. During the next 24 h the concentration of released virus increased more then 100-fold and was accompanied by a strong amplification of the phosphoprotein response. In order to validate the RPMA data, the levels of eight selected proteins were analyzed by Western blots using antibodies against phosphorylated and total forms of proteins (Fig. 4). Average band intensities from two independent Western blot experiments normalized to the total amount of cellular protein demonstrated good agreement with the data obtained from three independent RPMA experiments (Fig. 5).

**Table 1:** Signaling protein levels in RVFV-infected HSAECs.

Protein	48 h.p.i.						72 h.p.i.					
	Control		Low		High		Control		Low		High	
	M^1^	CI^2^	M	CI	M	CI	M	CI	M	CI	M	CI
Group I												
p-ERK	1.0	0.3	1.3	0.3	2.0	0.3	1.0	0.3	3.0	0.4	3.0	0.5
p-STAT1	1.0	0.3	1.6	0.1	4.0	0.3	1.0	0.2	4.9	0.5	4.7	0.6
Caspase 3 cleaved	1.0	0.4	1.3	0.1	1.9	0.4	1.0	0.2	3.3	0.2	3.5	0.3
Caspase 6 cleaved	1.0	0.1	1.1	0.0	1.9	0.2	1.0	0.1	2.6	0.1	3.0	0.3
Caspase 7 cleaved	1.0	0.1	1.2	0.1	1.8	0.4	1.0	0.0	3.0	0.2	2.9	0.1
p53 S15	1.0	0.1	2.5	0.2	5.6	0.3	1.0	0.2	8.6	1.1	5.6	0.5
p70S6 T412	1.0	0.1	1.2	0.1	1.5	0.1	1.0	0.0	1.8	0.3	1.3	0.1
Src Y416	1.0	0.1	1.0	0.1	1.2	0.1	1.0	0.1	1.2	0.1	1.4	0.1
Group II												
P53 S46	1.0	0.0	6.2	0.1	32	0.4	1.0	0.2	25	0.1	5.9	0.1
JNK T183/185	1.0	0.3	3.3	0.3	9.5	1.4	1.0	0.1	6.4	0.9	2.3	0.3
p90RSK S380	1.0	0.3	2.0	0.2	4.2	0.8	1.0	0.2	4.7	0.3	2.9	0.3
HSP27	1.0	0.1	2.1	0.3	4.5	0.5	1.0	0.2	2.3	0.1	1.8	0.2
MSK1 S360	1.0	0.4	2.0	0.2	3.6	2.2	1.0	0.7	1.6	1.2	1.5	0.5
p38 T180	1.0	0.2	1.9	0.2	3.8	0.2	1.0	0.2	3.8	0.3	2.2	0.4
MEK1/2	1.0	0.0	1.8	0.0	2.9	0.1	1.0	0.2	3.3	0.1	1.4	0.1
STAT3 S727	1.0	0.2	1.3	0.2	1.5	0.2	1.0	0.0	1.1	0.1	0.8	0.1
ATF2 T69/71	1.0	0.5	1.4	0.5	2.4	0.8	1.0	0.2	1.3	0.8	1.0	1.1
NF-kB S536	1.0	0.0	1.8	0.1	2.7	0.3	1.0	0.1	1.1	0.4	0.6	0.1
CREB S133	1.0	0.5	1.3	0.3	2.0	0.3	1.0	0.4	2.8	0.5	1.3	0.7
IRS S612	1.0	0.0	1.3	0.0	1.8	0.0	1.0	0.0	1.4	0.2	0.8	0.0
Src Y527	1.0	0.1	1.4	0.0	1.1	0.1	1.0	0.0	0.8	0.0	0.5	0.1
Group III												
AKT S473	1.0	0.1	1.1	0.1	0.8	0.1	1.0	0.2	0.6	0.2	0.5	0.2
XIAP	1.0	0.1	1.0	0.1	0.9	0.1	1.0	0.1	0.7	0.1	0.5	0.1
Bim	1.0	0.1	1.0	0.0	0.6	0.2	1.0	0.1	0.5	0.1	0.3	0.1
Puma	1.0	0.1	0.9	0.2	0.5	0.1	1.0	0.1	0.6	0.0	0.3	0.1
LC3b	1.0	0.2	1.0	0.1	0.9	0.1	1.0	0.1	0.8	0.2	0.5	0.2
PKR T451	1.0	0.1	1.1	0.1	0.9	0.1	1.0	0.1	0.8	0.1	0.6	0.1
FOX T24/31	1.0	0.1	1.0	0.0	0.9	0.0	1.0	0.0	0.8	0.0	0.8	0.1
Total protein levels												
ERK total	1.0	0.0	1.2	0.1	1.5	0.1	1.0	0.1	1.0	0.3	1.4	0.2
38 total	1.0	0.2	1.3	0.2	1.2	0.3	1.0	0.1	1.0	0.2	0.7	0.1
JNK total	1.0	0.2	1.2	0.2	1.1	0.1	1.0	0.0	1.2	0.1	1.0	0.1
AKT total	1.0	0.0	1.2	0.1	1.5	0.1	1.0	0.1	1.0	0.3	1.4	0.2
Actin	1.0	0.1	1.0	0.1	1.0	0.1	1.0	0.1	1.2	0.1	1.2	0.1

1 Arithmetic mean.

2 Confidence interval, Student's two-tail test, α = 0.05. RPMA data represent 3 independent experiments carried out in duplicate. The values are shown relative to the untreated HSAECs after normalization to the total cellular protein content at each time point.

All 27 signaling proteins can be divided into three groups according to similarities in their temporal phosphorylation patterns (Table 1). In the first group the phosphorylation of the mitogen-activated protein kinase ERK1/2 and transcription factor STAT1, which is activated through a p38 (mitogen-activated protein kinase) MAPK-dependent pathway in response to IFN-α and other cellular stresses, were constantly increased. The same pattern was demonstrated by the cleaved caspases-3, -6 and -7, which are major components of extrinsic and intrinsic apoptotic pathways. The second group includes 13 proteins comprising the transcription factors: p53 (S15 and S46) controlling cell cycle arrest, DNA repair or apoptosis; MSK-1 (S360) activated by ERK and p38; anti-apoptotic STAT3 (S727) activated through the MAPK or mTOR pathways; ATF2 (T69/71) activated by SAPK/JNK and p38 MAPK; NF-kB p65 (S536) playing a pivotal role in inflammatory and immune responses; and CREB (S133) mediating numerous cellular responses. Among Group II are kinases SAP/JNK (T183/Y185) and p38 (T180), which regulate various cellular activities such as gene expression, mitosis, differentiation, proliferation, and cell survival/apoptosis; MAPK kinase MEK1/2 (S217/221); the 90 kDa ribosomal S6 kinase, p90RSK (S380), which is activated *via* coordinated phosphorylation by MAPKs, phosphoinositide-3-OH kinase (PI3K) and autophosphorylation in response to many growth factors, polypeptide hormones and neurotransmitters; insulin receptor substrate IRS-1 (S612), which phosphorylation is necessary for activation of AKT; and HSP27, conferring cellular resistance to adverse environmental changes. For group II, protein phosphorylation demonstrated a transient pattern of initial increase followed by a decline detectable mostly at the MOI of 0.002. However, at lower MOI of 0.0002, no decline of phosphorylation took place for the majority of these 13 proteins, perhaps reflecting slower dynamics of the infection, compared to the higher MOI.

The third group of 7 proteins includes several protein kinases. Among them are serine/threonine kinase AKT (protein kinase B), the global regulator of survival; protein kinase R (PKR), which mediates apoptosis induced by viral double-stranded RNA (dsRNA); and the protein tyrosine kinase c-Src, which activity is regulated by tyrosine phosphorylation at two sites Y416 and Y527, but with opposite effects. Group III also includes apoptosis inhibitor XIAP. In this group the phosphorylation levels showed a statistically significant, continuous decrease during the infection. The same pattern of continuous decrease was demonstrated for the total level of autophagic protein LC3B, and the pro-apoptotic proteins Bim and Puma belonging to the Bcl-2 family.

### Analysis of pro-apoptotic signaling and caspase activation

**Figure 6:**
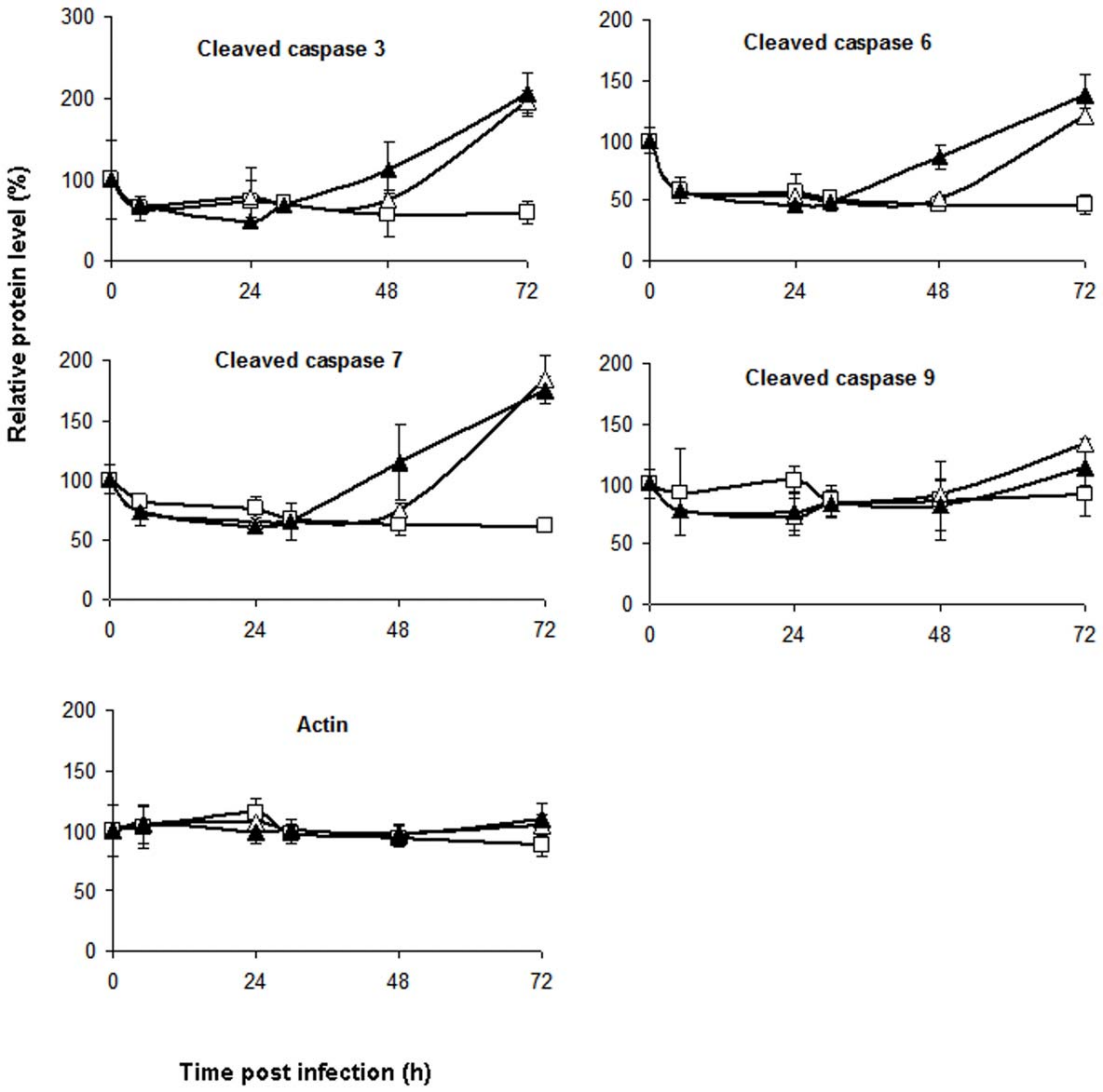
RPMA data for caspase-3, -6, -7, and -9 and actin. X axis labels, time post infection (h.p.i); Y axis labels, protein level relative to untreated cells (%) at 0 h.p.i. Open triangles, closed triangles and open squares correspond to the low MOI, high MOI and uninfected cells, respectively. Data represent mean values and confidence intervals of two-tail t-test (*α* = 0.05, *n* = 3).

Modulation of apoptosis is a known feature of RVFV [8]. Therefore, along with protein phosphorylation events we analyzed the levels of cleaved caspases. As indicated above, HSAECs infected with RVFV demonstrated increased levels of cleaved effector caspase-3, -6 and -7 at both MOIs; however, the level of caspase-9 cleaved at the activation sites D315 and D330 did not change significantly at both tested MOIs (Table 1, Fig. 6). The absence of significant changes in the level of caspase-9 involved in the intrinsic apoptotic pathway agrees with a large increase in the level of HSP27 protein, which is known to inhibit the apoptosome assembly and caspase-9 activation [17]. The levels of HSP70 and HSP 90, also capable of regulating the apoptosome, did not change in HSAECs infected with RVFV.

Further evidence in favor of the intrinsic apoptotic pathway inhibition by the infection was revealed by the behavior of the proapoptotic mitochondrial proteins of Bcl-2 family, namely: the Bcl-2-associated protein X (Bax); the p53-upregulated modulator of apoptosis (Puma); the pro-apoptotic (Bim); the Bcl-2-interacting domain (Bid); and Bad. The latter inhibits anti-apoptotic protein Bcl-XL and induces apoptosis when it is phosphorylated on S136 by AKT. In RVFV-infected cells, Bad (S136) phosphorylation demonstrated no substantial change until 72 h.p.i. The levels of Bid and Bax did not change, however a decrease was observed for Puma and Bim (Table 1). Collectively, these results are consistent with upregulation of extrinsic apoptotic signaling in infected cells.

### Analysis of mitogen-activated protein kinase (MAPK) signaling

MAPK (JNK, p38, ERK) pathways constitute a large kinase network that regulates a variety of physiological processes, such as cell growth, differentiation, and apoptotic cell death in response to different stimuli. We found that during RVFV infection in HSAECs the normalized levels of phosphorylation of MAPKs, including p38, JNK and ERK, were upregulated and followed specific patterns of changes (Table 1 and Fig. 5). As mentioned above, phosphorylation of ERK on T202/Y204 was steadily increased, although the total amount of ERK was relatively stable and even showed a tendency to decline at 72 h.p.i. in comparison with uninfected cells (Table 1 and Fig. 4, 5). In order to confirm activation of the ERK pathway, we tested the phosphorylation of MEK1/2 upstream of ERK1/2 with Western blotting (Fig. 4). As expected, transient phosphorylation of MEK1/2 on S217/S221 at 24 h.p.i. preceded activation of ERK, which took place at 48 h.p.i. (MOI of 0.002). Downstream from activated ERK1/2 the survival signaling was propagated to p90RSK (Group II in Table 1). This kinase can activate the transcription factor CREB, which promotes cell survival through transcriptional up-regulation of anti-apoptotic Bcl-2, Bcl-XL, and Bcl-1 proteins. Increased CREB phosphorylation on S133 was observed, in agreement with phosphorylation of p90RSK (Group II in Table 1).

JNK1/2 (c-Jun N-terminal kinase 1/2) pathway activity can mediate apoptosis, proliferation, or survival, depending on the stimuli and cellular conditions. Interestingly, sustained JNK1/2 activity is necessary for cellular homeostasis, whereas strong stress stimuli in non-transformed cells lead primarily to JNK-mediated apoptosis [18], [19]. Total amount of JNK1/2 did not change in comparison to untreated cells (Table 1, Fig. 4). Activated form of JNK1/2 phosphorylated on T183/T185 increased in infected HSAECs at 48 h.p.i. for both MOIs. This increase was followed by a strong dephosphorylation at MOI of 0.002, while JNK1/2 phosphorylation continued to increase at MOI of 0.0002 (Table 1 and Fig. 4, 5).

The p38 MAPK pathway can be activated in response to a plethora of inflammatory cytokines, as well as pathogens and by environmental stress, such as osmotic stress, ultraviolet light, heat shock, and hypoxia. The activation of the p38 MAPK pathway is required for apoptosis induction in several different cellular models [20]–[23]. Additionally, stress-elicited p38 activation was shown to cause G2/M cell cycle arrest and to regulate the cell cycle through modulation of p53 and p73 tumor suppressor proteins [24], [25]. Similar to the proteins from Group II (Table 1, Fig. 4, 5), RVFV infection in HSAECs stimulated phosphorylation of p38 on T180.

The activation pattern of MAPKs prompted us to analyze the phosphorylation of transcriptional factors as their most important substrates [26]. We found that the phosphorylations by RVFV of STAT1 (Y701), ATF2 (T69/71), MSK (S360), CREB (S133), NF-κB (S536), and p53 (S15/46) infection were transiently increased in general agreement with activation of MAPKs' phosphorylation (Table 1 and Fig. 4, 5).

### Downregulation of pro-survival signaling through decreased phosphorylation of pro-survival AKT and anti-apoptotic XIAP

The PI3K-AKT network activated by cytokines or growth factors mediates intracellular signals to regulate a variety of cellular responses, including anti-apoptosis, proliferation, cell cycling, protein synthesis, glucose metabolism and telomere activity [27]–[29]. RVFV infection decreased the phosphorylation of AKT on its major activation site S473 without considerable changes in the total amount of AKT in comparison with uninfected cells (Table 1 and Fig. 4). In agreement with this, the inhibitor of apoptosis, XIAP100 [30]–[33], was decreased in the infected cells, further contributing to apoptosis. Our data also show that RVFV infection downregulated the level of LC3B which is involved in cell autophagy.

### Effect of p38, JNK and MEK1/2 inhibitors on RVFV replication

Our results indicated that MAPK pathways regulating cell fate in response to different stimuli were activated during RVFV infection. Therefore, we were interested in determining if inhibitors of these pathways could affect RVFV replication. For this purpose, we chose to test the inhibitors of p38 (SB203580), JNK (JNK Inhibitor II) and ERK1/2 (MEK1/2 Inhibitor III) in cells infected with RVFV MP-12. In preliminary experiments we showed that the MP-12 strain induced ERK1/2, p38, and JNK phosphorylation in a manner similar to ZH-501 (Fig. S1). We also found that in agreement with the ZH-501 data, the MP-12-infected cells demonstrated the onset of apoptosis evident in the increased degradation of cellular DNA. Figure S2 shows progressive accumulation of cells in the sub-G1 phase of cell cycle characteristic of cell death during the course of infection.

**Figure 7:**
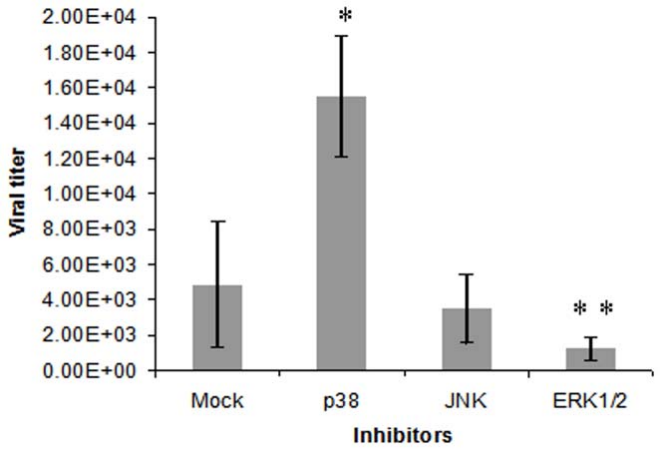
Effect of inhibitors on RVFV replication. HSAECS were plated at 5×10^4^ cells per well in a 96-well plate and pretreated with either DMSO or indicated inhibitors for 5 h prior to RVFV infection at MOI of 0.002 as described in Materials and Methods. Following infection, fresh medium containing inhibitors was added. Supernatants were collected at 48 h.p.i., viral RNA extracted and analyzed by q-RT-PCR. * p = 0.0002, ** *p* = 0.031 (t-test) relative to mock control.

HSAECs pretreated with the inhibitors for 5 h were infected with RVFV MP-12 at MOI 0.002. Supernatants were collected at 48 h.p.i. and analyzed by q-RT-PCR to determine the influence of these inhibitors on viral replication. We found that the cells treated with SB203580 demonstrated increased viral release (*p* = 0.0002) compared to the untreated cells (Fig. 7). The inhibitor of JNK did not show a statistically reliable change, indicating that JNK pathway might not control viral replication. However, the MEK1/2 inhibitor selectively affecting phosphorylation of the downstream ERK1/2 decreased viral titer (*p* = 0.031). No cell toxicity was observed at the concentrations of inhibitors used, indicating that the decrease in viral replication was not due to cell death (data not shown). These results suggested that p38 activity represents the protective cell response while activation of ERK1/2 pathway contributes to viral replication. Mechanisms of these effects are a subject of ongoing investigation.

## Discussion

Host cell signaling is considered as one of the targets for future antiviral therapies. However, very little information is currently available regarding the RVFV infection in susceptible cells relevant to inhalation exposure. We chose experimental conditions with low MOI, likely reflecting the low-dose exposure to the virus in the natural environment, and found the lung epithelial cells to be highly susceptible. The cytopathic changes in the cells exposed to virulent strain ZH-501 took place over the period of 72 h, which allowed us to monitor a gradual development of infection, with the focus to identify the major pathways involved in the regulation of cell death.

**Figure 8:**
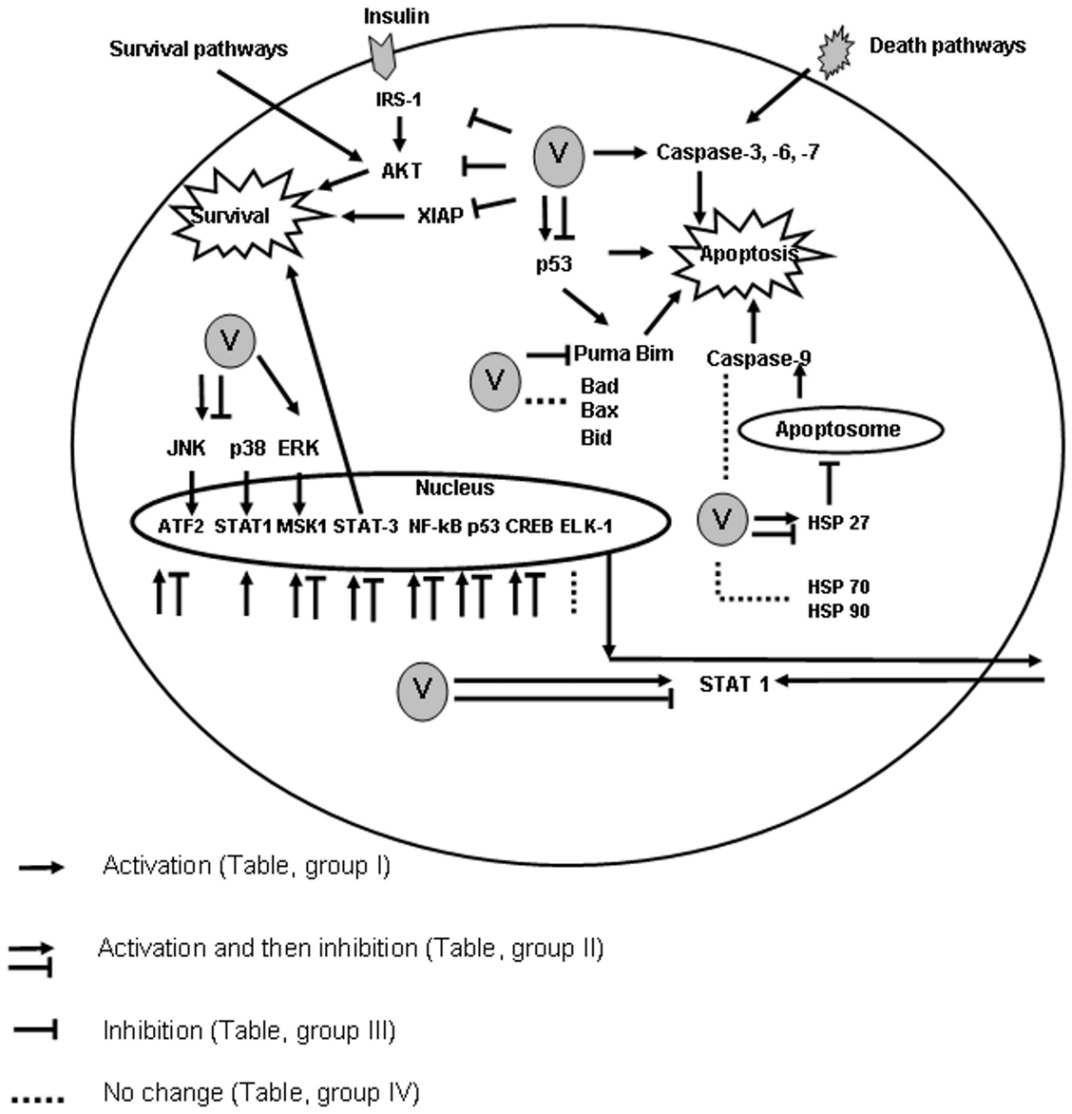
RPMA data presented as a diagram of signaling events in HSAECs infected with RVFV. Among the most prominent responses was activation of MAP kinases (p38, JNK and ERK) and the downstream transcriptional factors [STAT1 (Y701), ATF2 (T69–71), MSK1 (S360), and CREB (S133)]. STAT3 and NF-κB phosphorylation were also increased. Activation of p53(S15, S46), which typically accompanies transcriptional upregulation of the apoptotic genes correlated with the increased levels of cleaved effector caspase-3, -6 and -7 indicating activation of the extrinsic apoptotic pathway. RVFV infection also downregulated phosphorylation of a major anti-apoptotic regulator of survival pathways, AKT (S473). Consistent with this, the level of apoptosis inhibitor XIAP100 was decreased. The intrinsic apoptotic pathway marker, caspase-9, demonstrated only a marginal activation accompanied by increased level of the inhibitor of apoptosome formation, HSP27.

In our study for the first time we undertook quantitative analysis of phosphoprotein signaling induced by RVFV in HSAECs using RPMA. This approach allowed us to analyze at the same time the changes in the levels of phospho-modifications, activated forms, or total levels for 60 proteins. The signaling pathways altered in HSAECs infected with RVFV are summarized in Fig. 8. Significant changes in PI3K-AKT, MAPK, apoptotic and p53 pathways were observed. The AKT pathway is known to be one of the prominent anti-apoptotic programs in mammalian cells that leads to inactivation of numerous apoptotic stimuli [27]–[29]. PI3K-Akt pathway is an important mechanism through which viral infection influences various cell functions. Activating PI3K-AKT signaling can slow down apoptosis and prolong viral replication in both acute and persistent infection [reviewed in ref. 34]. Accumulating evidence suggests that the activity of PI3K or AKT is critical for survival of several viruses. Adenovirus relies on PI3K-mediated organization of actin filament for active internalization. Non-segmented negative-sense RNA viruses require AKT to enhance synthesis of viral RNAs. On the other hand, PI3K-AKT signaling is associated with up-regulating interferon response. Higher PI3K-AKT activity might impede viral propagation due to activation of cellular defenses. Influenza A virus requires active PI3K for penetration despite the negative effects of inducing immune response. Unlike most viruses, it was reported that VP1 protein of foot-and-mouth disease virus inhibits AKT to promote cell death [reviewed in ref. 34] RVFV inhibition of AKT phosphorylation in HSAECs can promote cell death. Identification of virus protein(s) inducing this anti-survival signaling may provide clues to virus pathogenicity.

The conserved inhibitor of apoptosis (IAP) family plays an important role in regulation of apoptosis through modulation of caspases [32], [33]. IAPs can bind directly to caspases, leading to either their inactivation or degradation. The most potent caspase inhibitor of the IAP family is X-linked IAP, XIAP [33], [35]. In the RVFV-infected HSAECs, AKT and XIAP downregulation as well as the caspase upregulation are progressively increased and therefore likely predispose cells to apoptosis.

Caspases -3, -6 and -7 are effector caspases for both the intrinsic and extrinsic apoptotic pathways. To distinguish between these apoptotic pathways we analyzed proteins upstream of effector caspases. The cysteine aspartyl protease caspase-9 plays a central role in the mitochondrial or intrinsic apoptotic pathway that is engaged in response to many apoptotic stimuli. Caspase-9 is activated in a large multimeric complex, the apoptosome, which is formed with apoptotic peptidase activating factor 1 (Apaf1) in response to the release of cytochrome C from mitochondria [36]. Cleavage of caspase-9 on two sides Asp315 and Asp330 are necessary for there activation. Once activated, caspase-9 cleaves and activates the effector caspases 3 and 7 to bring about apoptosis. This pathway is tightly regulated at multiple steps, including apoptosome and cleaved caspase-9 formation. We detected no statistically significant changes in the level of cleaved caspase-9 between uninfected and RVFV-infected HSAECs. Therefore activation of caspases -3 and -7 most likely took place through the extrinsic pathway. Increased level of HSP27 is consistent with this conclusion [30]. In this respect, manipulation with caspases is not unique to RVFV. Influenza virus was reported to induce apoptosis by extrinsic death signaling through Fas/Fas-associated death domain-containing protein (FADD)/caspase-8 pathway [37]. The level of cellular caspase-3 activity correlated directly with the amount of progeny virus produced in the host cell line infected with influenza virus [38]. Similarly, the inhibition of caspases 3 and 9 with synthetic inhibitors, along with reducing apoptosis, reduced infective adenovirus titer in a dose-dependent manner [39].

p53 acts predominantly as a transcription factor, regulating the expression of more then 100 target genes to initiate apoptosis, cell cycle arrest, DNA repair, cellular senescence as well as differentiation [40]. RVFV induced the increase in phosphorylation of p53 on both apoptosis-related sides, on S15 and S46. However, our data do not show increased levels of apoptotic proteins transcriptionally regulated by p53 (Bax, Bad, Bim, Puma, and Bid). This indicates that RVFV might possess other mechanisms of apoptosis downregulation through the intrinsic pathway, such as the NSm-dependent one [8], in addition to the upregulation of HSP27 discussed above.

Members of the MAPK family are involved in signal transduction of apoptosis as well as cell growth and differentiation. During RVFV ZH-501 infection in HSAECs the phosphorylations of MAPKs including p38, JNK and ERK were found to be upregulated. ERK1/2 plays a central role in cell proliferation and differentiation [41]–[43]. At both MOIs, constant progression of ERK1/2 activation took place in HSAECs infected with RVFV in agreement with the increased phosphorylation on S380 of the upstream ERK1/2-activating kinase p90RSK. Activation of p90RSK was also evident in the increased phosphorylation of the downstream CREB. This pattern was consistent with a pro-survival host response through ERK1/2, although it did not exclude the role of the ERK signaling cascade in promoting RVFV replication, as it has been shown for influenza virus [44].

The JNK pathway is activated by cellular stress and by cytokine induction. JNK pathway activity can mediate apoptosis, proliferation, or survival, depending on the stimuli and cellular conditions. Sustained JNK activity is necessary for cellular homeostasis, whereas strong stress stimuli in non-transformed cells primarily lead to JNK-mediated apoptosis. The activated form of JNK phosphorylated on T183 and Y185 was increased in RVFV-infected HSAECs initially and then dramatic dephosphorylation was observed (more then 10-fold at 72 h.p.i. in comparison with 48 h.p.i) by the higher dose of infection, indicating that it is one of the major RVFV targets.

Activation of the p38 MAPK pathway is required for apoptosis induction in several different cellular models [20]–[23]. Additionally, stress-elicited p38 activation was shown to cause G2/M cell cycle arrest and to regulate the cell cycle through modulation of p53 and p73 tumor suppressor proteins [24] RVFV infection in HSAECs demonstrates similar patterns of phosphorylation for p38 and JNK for both of these pathways important for cell survival. Phosphorylated p38 proteins can activate an array of transcription factors, including ATF2, CHOP1, MEF2, p53, STAT1 and Elk1. For JNK a classical substrate is the transcription factor c-Jun along with ATF2, Elk1, MEF2c, p53, STAT1, and c-Myc. Our data for STAT1 (Y701), ATF2 (T69–71) and MSK1 (S360) indicate their coordinated upregulation with MAPKs and implicate RVFV in a widespread control of cellular transcription.

Taking into account the importance of MAPK pathways for cell fate we tested the effect of p38, JNK and ERK1/2 phosphorylation inhibitors on replication of RVFV MP-12 strain, which demonstrated activation of MAPKs similar to ZH-501. Using the inhibitor-pretreated cells, we found that viral growth can be promoted or inhibited by the p38 and ERK1/2 pathway inhibitors, correspondingly, while JNK cascade did not exert a strong effect on the amount of released virus. These data support a potential utility of phosphoprotein signaling cascades as host targets for treatment of viral infections. However, a strong influence of MOI on the level and dynamics of transient cell responses (Table 1) brings up an additional level of complexity, which needs to be addressed in future studies. For example, phosphorylation of several proteins from group II at certain time demonstrated either increasing or decreasing trends, depending on the progress of infection. We therefore suggest that potential signaling-based therapeutic interventions would need to take into account not only the nature but also a magnitude of the particular response for adequate treatment. Future experiments with specific inhibitors administered at different times post infection will help understand the significance of the identified pathways for survival/death cell decision in RVFV-infected HSAECs. The experiments with aerosol RVFV-infected animals are also forthcoming to analyze virus-induced signaling in the lung and to reconcile these findings with our *in vitro* model.

## Supporting Information

Figure S1 Induction of ERK1/2, p38, and JNK by RVFV MP-12. Western blots were performed on lysates of RVFV MP-12-infected HSAECs at indicated times post infection with MOI of 0.002 (for ERK1/2, p38 and JNK). Open and grey bars show band intensities of uninfected and infected cells, correspondingly, calculated relative to uninfected cells at 24 h.p.i.
(0.92 MB TIF)

Figure S2 Sub-G1 cell cycle fraction of HSAECs is increased during RVFV infection. HSAECs were mock infected or infected with RVFV MP12 at MOI of 3.0. Cells were collected at 6, 24, and 48 h.p.i. and cell cycle analysis performed by propidium iodide staining. Flow cytometry data were acquired on a Becton Dickinson FACScaliber with a 488-nm argon laser. Acquisition and analysis were performed with CELLQuest software. A. The percent of the sub-G1 population relative to the total number of cells as average of two independent samples. Open bars, mock-infected cells; gray bars, RVFV-infected cells. B. Histogram of the sample at 48 h.p.i. illustrating the position of the sub-G1 fraction gate.
(0.71 MB TIF)
