# Supplementary figures and images for: Reverse-Phase Phosphoproteome Analysis of Signaling Pathways Induced by Rift Valley Fever Virus in Human Small Airway Epithelial Cells
Source: PLoS One. 2010 Nov 3;5(11):e13805. doi: 10.1371/journal.pone.0013805 (PMC2972203; doi:10.1371/journal.pone.0013805)

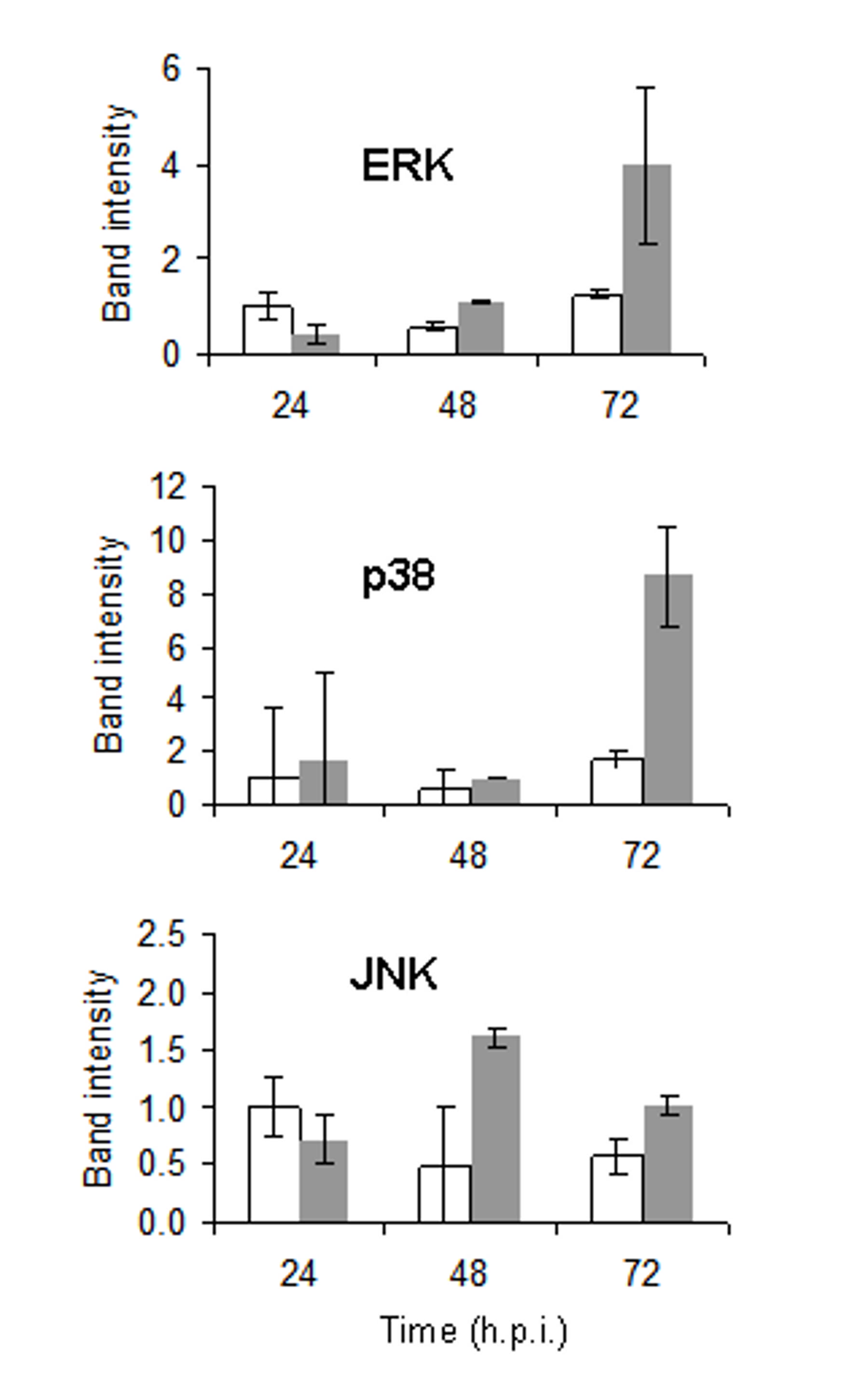

Supplement: Figure S1 — Induction of ERK1/2, p38, and JNK by RVFV MP-12. Western blots were performed on lysates of RVFV MP-12-infected HSAECs at indicated times post infection with MOI of 0.002 (for ERK1/2, p38 and JNK). Open and grey bars show band intensities of uninfected and infected cells, correspondingly, calculated relative to uninfected cells at 24 h.p.i. (0.92 MB TIF) [file pone.0013805.s001.tif]

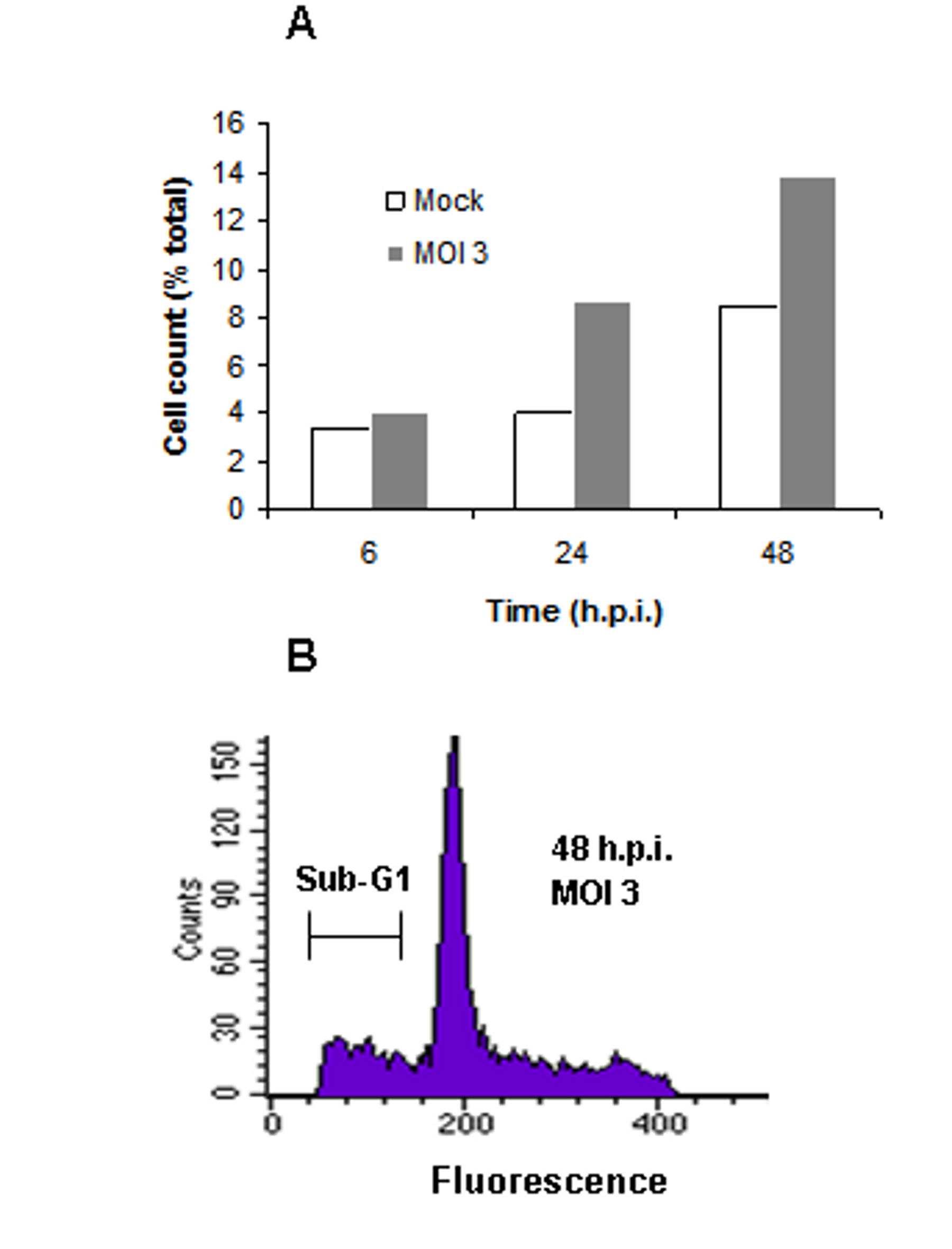

Supplement: Figure S2 — Sub-G1 cell cycle fraction of HSAECs is increased during RVFV infection. HSAECs were mock infected or infected with RVFV MP12 at MOI of 3.0. Cells were collected at 6, 24, and 48 h.p.i. and cell cycle analysis performed by propidium iodide staining. Flow cytometry data were acquired on a Becton Dickinson FACScaliber with a 488-nm argon laser. Acquisition and analysis were performed with CELLQuest software. A. The percent of the sub-G1 population relative to the total number of cells as average of two independent samples. Open bars, mock-infected cells; gray bars, RVFV-infected cells. B. Histogram of the sample at 48 h.p.i. illustrating the position of the sub-G1 fraction gate. (0.71 MB TIF) [file pone.0013805.s002.tif]
